# Cellular therapy in neuromyelitis optica spectrum disorder

**DOI:** 10.3389/fneur.2025.1709474

**Published:** 2025-11-24

**Authors:** Kang Zhong, Yulan Tang

**Affiliations:** Department of Neurology, First Affiliated Hospital of Guangxi Medical University, Nanning, China

**Keywords:** neuromyelitis optica spectrum disease (NMOSD), autologous hematopoietic stem cells (AHSC), chimeric antigen receptor T cells (CAR-T), bone marrow-derived mesenchymal stem cells (BM-MSCs), human umbilical cord-derived mesenchymal stem cells (hUC-MSCs)

## Abstract

Neuromyelitis optica spectrum disorder (NMOSD) is a debilitating neuroimmune condition characterized by recurrent inflammatory episodes that result in progressive disability, substantial psychological distress, and significant economic burdens. While current immunosuppressive therapies reduce relapse rates, they fail to achieve a definitive cure. Cellular therapy has emerged as a novel therapeutic strategy, offering potential disease modification through immune cell modulation and immune system reorganization via *in vivo* transplantation of adult stem cells or genetically engineered somatic cells. This review synthesizes recent advancements in cellular therapy for NMOSD, aiming to improve understanding of it in NMOSD disease and provide a roadmap for future research.

## 1 Introduction

Neuromyelitis optica spectrum disorder (NMOSD), first described by Eugène Devic in 1894 and historically termed Devic’s disease, is a neuroimmune demyelinating disorder characterized by recurrent inflammatory attacks predominantly targeting the optic nerves and spinal cord (1). The prevalence of this disease exhibits considerable geographic variation, being highest in Africa and lowest among Caucasian populations. Incidence rates also differ across age groups, with adult prevalence ranging from 0.34 to 10 per 100,000, compared to 0.06 to 0.22 per 100,000 in pediatric populations (2). Notably, the female-to-male prevalence ratio ranges from 2.3 to 7.6 times higher in women among both Caucasian and African ethnic groups. Regional disparities in mortality rates have been documented, though comprehensive global studies remain unavailable (3, 4). Relapses of the disease lead to cumulative neurological disability, severely compromising patients’ quality of life and imposing profound socioeconomic burdens on affected individuals and their families, even increasing the risk of death (4, 5). Current therapeutic strategies rely on immunosuppressive agents such as azathioprine, mycophenolate mofetil, rituximab, inebilizumab, and satralizumab to mitigate relapse frequency (6, 7). However, these treatments fail to achieve complete remission, with observational studies reporting that nearly 50% of NMOSD patients experience one or more relapses despite adherence to immunosuppressive regimens (8). Current medications only suppress the immune system, target specific pathological pathways, or decrease the production of pathogenic antibodies; however, they do not inhibit the disease at its origin (9). Consequently, there is a need to develop drugs or modulatory therapies that can target the very early stages of the disease, which could potentially lead to a cure for individuals at the biological stage of the disease or offer preventive treatment for those at risk. Furthermore, approaches that can restore the dysregulated immune system are also necessary.

**Figure 1:**
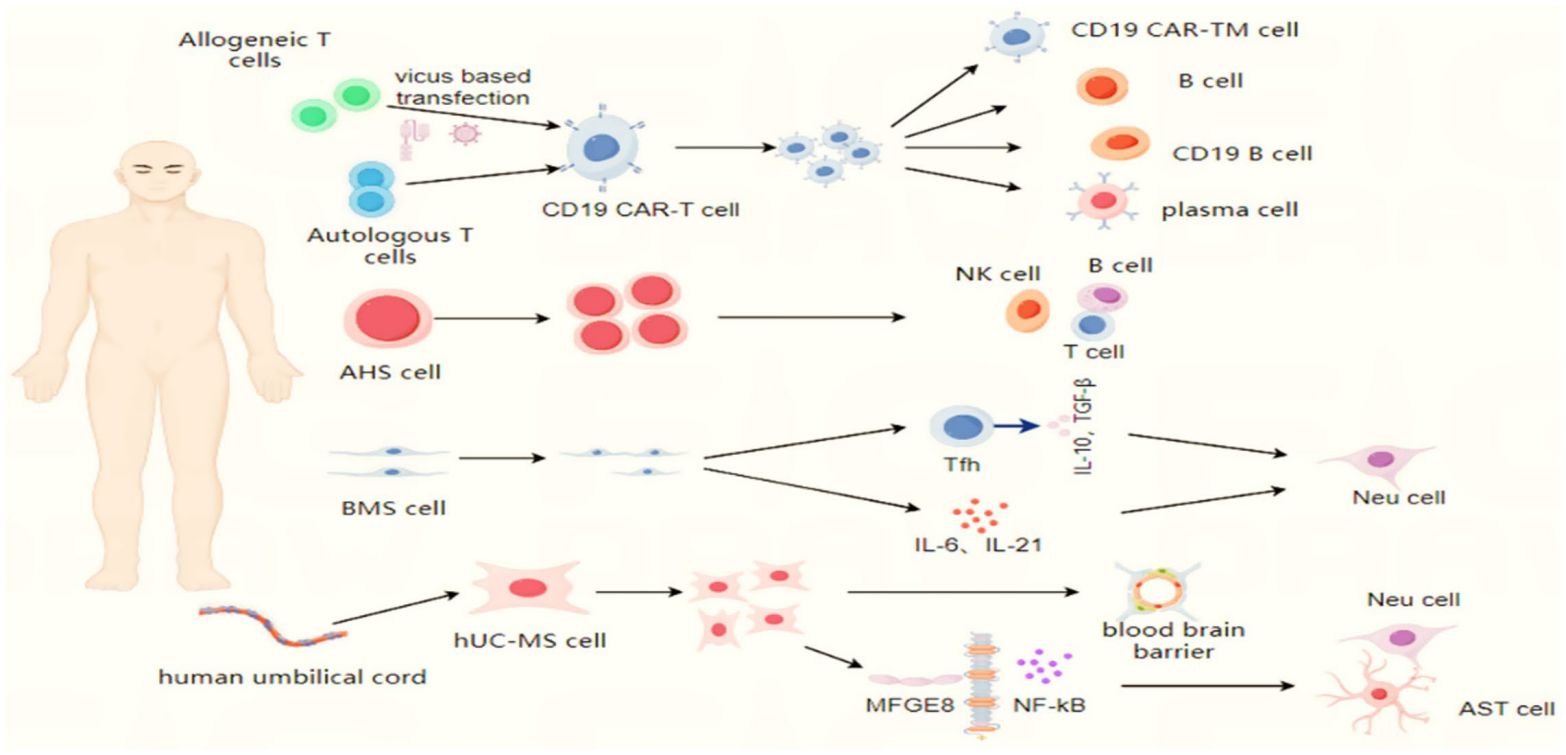
The mechanism of action of different cell therapies in neuromyelitis optica spectrum disorder. T lymphocytes derived from allogeneic or autologous sources are genetically modified to become CD19-targeting T cells (CAR-T). Following ex vivo expansion, these engineered cells are reinfused into the patient, where they specifically identify and eliminate CD19-positive B lymphocytes and antibody-secreting plasma cells. A subset of these cells further differentiates into memory T lymphocytes, thereby mediating long-term immunosurveillance. Autologous hematopoietic stem cells are reinfused into the myeloblast host, where they differentiate into new immune cells and reconstitute the immune system. Bone marrow-derived mesenchymal stem cells exert neuroprotective effects via the secretion of inflammatory cytokines such as IL-6 and IL-21, and/or through the modulation of T follicular helper (Tfh) lymphocytes. Human umbilical cord-derived mesenchymal stem cells act on astrocytes to ameliorate blood–brain barrier injury. Additionally, they secrete MFGE8, which inhibits immune-induced NF-κB activation and reduces the release of pro-inflammatory factors, thereby attenuating inflammatory infiltration and protecting motor neurons from damage.

Cellular therapy has emerged as a promising frontier in immune-mediated disorders (10). Adult stem cells—including hematopoietic stem cells (HSCs), mesenchymal stem cells (MSCs), and neural stem cells—have demonstrated therapeutic potential in preclinical and clinical studies of autoimmune diseases (11–14). For instance, HSC transplantation and MSC infusion have shown efficacy in resetting dysregulated immune responses and promoting tissue repair in multiple sclerosis (15, 16). Autologous hematopoietic stem cell transplantation has shown a higher rate of disability improvement in the treatment of active relapsing–remitting multiple sclerosis and is more effective in preventing relapses compared to other immune-modulating treatments (17–19). Also, genetically modified chimeric antigen receptor T cells, utilizing gene editing techniques, have achieved impressive results in the treatment of the disease, providing hope for a cure for patients suffering from immune disorders (20–22). Building on these advances, this review synthesizes recent progress in cellular therapy for NMOSD, with a focus on clinical applications and challenges (see Figure 1).

## 2 Adult stem cell therapy

### 2.1 Autologous hematopoietic stem cells

Hematopoietic stem cells play a crucial role in disease treatment due to their ease of acquisition and well-established extraction and preservation techniques. Based on their source, hematopoietic stem cells can be classified as autologous or allogeneic. Hematopoietic stem cell transplantation (HSCT) is a therapeutic approach that involves removing abnormal cells from the body through radiotherapy or chemotherapy, followed by the infusion of the patient’s own or another person’s hematopoietic stem cells to restore normal hematopoietic and immune functions. In this manner, the disease can be cured by eliminating defective inflammatory responses and immune cells from the autoimmune system and establishing a self-tolerant immune system. Autologous hematopoietic stem cells are popular for many diseases due to the absence of autoimmune rejection.

In multiple sclerosis (MS), HSCT has been demonstrated to be effective in preventing relapses and reducing disease severity in a relatively safe and cost-effective manner (18, 23). A follow-up study found that HSCT treatment improved disability, reduced relapse rates, and thus enhanced the quality of life in patients with NMOSD (24). The conversion to negative AQP4 antibodies after treatment may indicate that patients can remain free of immunosuppressive drugs for an extended period, potentially related to the immune reconstitution of the organism. AHSCT is capable of reconstituting the repertoire of self-tolerant T and B lymphocytes, thereby rebuilding the immune system and altering the inflammatory milieu (25). However, the precise characteristics of immune reconstitution following AHSCT in patients with NMOSD remain poorly characterized. Given the remodeling role of AHSCT in immune disorders, Koh Yeow Hoay proposed that AHSCT could serve as an alternative therapy to conventional immunotherapy in patients with severe NMOSD, providing an option for those who have difficulty receiving conventional immunotherapy (26). Autologous peripheral hematopoietic stem cell transplantation may also contribute to reducing the frequency of neuromyelitis optica attacks (27). However, unlike malignant hematologic diseases, NMOSD may recur at a later time point despite transient remission after AHSCT treatment for NMOSD, which aligns with the description provided by Joachim Burman et al. (28, 29). AHSCT treatment rebuilds the immune system and offers hope for curing the disease, but the presence of complications should not be overlooked, such as fever, infection, hematopenia, and secondary autoimmune diseases (30–32). Similar complications can be observed in multiple sclerosis (33). Meanwhile, most of the current studies on the role of AHSCT in the treatment of NMOSD have been conducted in adults, and the therapeutic value in pediatric NMOSD remains to be further determined (34–36). These factors restrict the broader utilization of hematopoietic stem cells in clinical settings. Overall, the effectiveness of AHSCT therapy in patients with NMOSD represents a therapeutic alternative worthy of consideration, particularly for patients experiencing recurrent or refractory NMOSD (26, 32, 37, 38). Future studies will focus on large-sample, multicenter data collection and clarifying the therapeutic dose of hematopoietic stem cells in NMOSD.

### 2.2 Mesenchymal stem cells

Mesenchymal stem cell (MSC) transplantation involves the isolation of MSCs from donor tissues, followed by *in vitro* expansion, purification, and subsequent reintroduction into patients to modulate disease pathology. As multipotent stromal cells, MSCs exhibit robust differentiation capacity, self-renewal potential, and paracrine activity mediated through cytokine secretion, T lymphocytes to suppress proliferation, and extracellular vesicle release (39–41). Critically, MSCs demonstrate low immunogenicity due to minimal MHC class I expression, alongside advantages such as abundant tissue sources (e.g., bone marrow, umbilical cord, placental tissue, adipose) and absence of ethical controversies, rendering them widely utilized in regenerative and immunomodulatory therapies (42, 43). These properties position MSC-based therapies as a promising strategy for immune reconstitution in neuromyelitis optica spectrum disorder (NMOSD), particularly in recalcitrant cases unresponsive to conventional immunosuppression.

#### 2.2.1 Human umbilical cord- derived mesenchymal stem cells

Emerging evidence suggests that human umbilical cord-derived mesenchymal stem cells (hUC-MSCs) exhibit therapeutic potential in ameliorating clinical signs and symptoms of NMOSD without reported adverse effects (44). A 10-year longitudinal follow-up study further corroborated the safety and feasibility of combined intravenous and intrathecal (IT) administration of hUC-MSCs, with no severe treatment-related complications documented (45). Short-term clinical evaluations have additionally confirmed the efficacy and favorable safety profile of hUC-MSCs in managing severe NMOSD cases (46). A prospective multicenter randomized placebo-controlled trial systematically investigated hUC-MSC-based therapy for NMOSD, providing robust evidence for its clinical safety and therapeutic efficacy while establishing critical groundwork for optimizing stem cell dosing protocols (47). In the animal model of NMOSD, treatment with hUC-MSCs was observed to improve dyskinesia by inhibiting astrocyte injury mediated by AQP4 antibodies and complement, attenuating inflammatory infiltration of the spinal cord, and reducing damage to the blood–brain barrier (48). Furthermore, hUC-MSCs secrete MFGE8, which interacts with the ITGB3 receptor on astrocytes. This ligand-receptor binding inhibits immune-induced NF-κB activation and reduces the release of pro-inflammatory factors, thereby protecting motor neurons from damage and ameliorating motor deficits (49).

#### 2.2.2 Bone marrow marrow-derived mesenchymal stem cells

Bone marrow serves as a primary source of mesenchymal stem cells (BM-MSCs), which have demonstrated therapeutic efficacy in hematologic disorders. In the NMOSD, BM-MSC therapy may reduce disease relapse rates and mitigate neurological disability. A two-year prospective observational study established that BM-MSC administration promotes structural restoration of the optic nerve and spinal cord in NMOSD patients, with sustained clinical benefits observed during follow-up (50). Mechanistic studies suggest that BM-MSCs mediate neuroprotection via immunomodulatory effects on T follicular helper (Tfh) cells and suppression of pro-inflammatory cytokines, including IL-6 and IL-21. Intriguingly, systemic absorption of MSCs through pressure ulcer sites has been proposed as an alternative delivery mechanism (51). However, BM-MSCs isolated from NMOSD patients exhibit intrinsic limitations, characterized by reduced proliferative capacity and accelerated senescence compared to healthy donor-derived counterparts (52). Furthermore, allogeneic BM-MSCs lack immune privilege and may trigger both humoral and cellular immune responses, potentially leading to treatment-related complications (53). These constraints highlight challenges in optimizing BM-MSC-based therapies for NMOSD. Exosomes derived from BM-MSCs represent a promising therapeutic strategy. Studies demonstrate their efficacy in promoting neural stem cell proliferation in spinal cord ischemia–reperfusion injury models (54). Moreover, in experimental autoimmune encephalomyelitis (EAE) models, they ameliorate central nervous system inflammation and demyelination, potentially via modulating microglial polarization or directly targeting oligodendrocyte precursor cells. Nevertheless, their therapeutic potential for NMOSD requires further investigation (55).

#### 2.2.3 Limitations of mesenchymal stem cell therapy

Despite the therapeutic potential of mesenchymal stem cells (MSCs) in NMOSD, the translation of laboratory findings to clinical applications encounters considerable challenges, including immunocompatibility, stability, heterogeneity, differentiation capacity, and migratory efficiency (56). Systemic infusion of MSCs may elicit severe adverse effects, such as thrombosis and embolism, especially with MSCs derived from non-bone marrow sources (57). To date, limited data are available on MSC-related adverse events in NMOSD treatment, and most studies have focused on umbilical cord- and bone marrow-derived MSCs. Drawing inspiration from CAR-T cell technology, Olivia Sirpilla et al. proposed that chimeric antigen receptor-engineered MSCs (CAR-MSCs) demonstrate enhanced immunosuppressive efficacy in murine models by upregulating immune checkpoint genes, T-cell inhibitory receptors, and immunosuppressive cytokines, without compromising cellular phenotype or safety profiles (58). This innovative approach may overcome current limitations and open new avenues for clinical translation of MSC therapies in neuroinflammatory disorders. Overall, MSC therapy exhibits promising efficacy, safety, and stability in the management of NMOSD, with umbilical cord-derived MSCs (UC-MSCs) demonstrating comparative advantages in proliferation capacity and immunomodulatory properties.

## 3 Chimeric antigen receptor T-cell therapy

CAR-T cell therapy involves the genetic engineering of autologous or allogeneic T cells through the design of chimeric antigen receptor (CAR) structures, selection of viral vectors, and transduction *in vitro*, followed by reinfusion into patients to treat disease (59, 60). While this approach has demonstrated remarkable efficacy in oncology, its application in autoimmune disorders like NMOSD is emerging (59, 61). In contrast to the immune reconstitution achieved by hematopoietic stem cells or the indirect immunomodulatory effects of mesenchymal stem cells, CAR-T cells can effectively cross the blood–cerebrospinal fluid barrier, directly act on pathological sites within the central nervous system, eliminate B cells in the cerebrospinal fluid, and reduce the production of autoantibodies, thereby suppressing neuroinflammation. Importantly, engineered CAR-T cells enable precise targeting of CD19-positive cells and eradicate antibody-producing plasma cells, achieving a targeted therapeutic strategy (62).

Notably, CAR-T cells exhibit potent *in vivo* expansion and generate memory T-cell populations, enabling sustained therapeutic effects—a potential foundation for disease remission (21, 63). In relapsed/refractory NMOSD patients unresponsive to conventional immunosuppression, CAR-T therapy demonstrates controllable safety and promising efficacy. A dose of ≤1.0 × 10^6^ CAR-T cells/kg has shown clinical stability and unexpected improvements in coexisting autoimmune conditions (64, 65). However, treatment-related toxicities, including cytokine release syndrome (CRS) and immune effector cell-associated neurotoxicity syndrome (ICANS), remain significant concerns (65). Evidence on CAR-T efficacy in CNS-targeted autoimmunity is sparse, with only anecdotal reports of individual benefits (66). However, the mechanisms of action of CAR-T cells in the treatment of NMOSD remain to be fully elucidated. Post-infusion infections (particularly within 28 days) correlate with CRS severity, mirroring observations in hematologic malignancies (67). Theoretical risks of CAR-T-induced oncogenesis necessitate long-term surveillance (68). Prohibitive pricing (€307,200–€350,000 per treatment) restricts accessibility for most patients (69). While CAR-T therapy represents a paradigm shift in NMOSD management, multicenter trials with larger cohorts are urgently needed to optimize dosing, mitigate neurotoxicity, and validate cost-effectiveness (see Tables 1, 2).

**Table 1:** Comparison of different cell therapies in NMOSD.

Feature	Autologous hematopoietic stem cell transplantation (AHSCT)	Mesenchymal stem cell transplantation (MSCT)	CAR-T cell therapy
Mechanism of action	Immune system reset: Uses high-dose chemotherapy/radiotherapy to eliminate abnormal immune cells in the patient’s body. Reinfuses pre-collected AHSCT to rebuild a new, potentially self-antigen tolerant immune system.	Immunomodulation and tissue repair: Modulates the function of immune cells (e.g., T cells, B cells) and suppresses inflammation via cytokine secretion (e.g., MFGE8) and cell-to-cell contact. Neuroprotection: Reduces astrocyte injury, blood–brain barrier disruption, and demyelination; promotes repair.	Precise elimination: Targets and eliminates B cells producing pathogenic antibodies (e.g., AQP4-IgG), such as CD19 + B cells. Can cross the blood–brain/blood-CSF barrier, acting directly within the central nervous system.
Therapeutic goal	Destroy and rebuild the entire aberrant immune system.	Modulate the overall immune environment and repair damaged neural tissue.	Eliminate specific pathogenic cells (B cells).
Main advantages	A mature method supported by long-term follow-up data. May achieve long-term disease-free survival for severe, refractory patients unresponsive to conventional therapy. May lead to AQP4 antibody seroconversion (negative) and allow discontinuation of immunosuppressants	Relatively high safety profile and low immunogenicity. Pleiotropic effects: Combines dual actions of immunomodulation and neural repair. HUC-MSCs: Sourcing is convenient, proliferation capacity is strong, ethical concerns are fewer; potentially more advantageous than BM-MSCs.	Precise targeting and direct mechanism of action. Strong *in vivo* expansion capability, potentially leading to long-term, sustained efficacy (due to memory T cells). Early studies show significant potential for refractory patients, possibly even achieving “functional cure.”
Limitations and risks	High treatment-related risks: Severe infections, bleeding, organ toxicity, treatment-related mortality. Potential for relapse: Possible recurrence even after immune reconstitution. Complications: Secondary autoimmune diseases, secondary malignancies. Value in pediatric NMOSD patients needs further determination.	Heterogeneous and potentially transient efficacy: Variable effects between cell batches, may require multiple infusions. BM-MSC Limitations: BM-MSCs from NMO patients may have decreased proliferation rates and susceptibility to senescence; allogeneic transplantation might trigger immune reactions. Migration & Survival: In vivo migration, survival time, and precise mechanisms require further clarification. Potential risks: Thrombosis, embolism.	Toxicities: Cytokine Release Syndrome (CRS), Immune Effector Cell-Associated Neurotoxicity Syndrome (ICANS). Long-term immunosuppression: B-cell depletion may increase infection risk. Extremely high cost. Limited data; mechanisms of action and long-term safety require more research.
Clinical application stage	Relatively mature therapy, used for severe patients with poor prognosis, high disease activity, refractory to conventional treatment.	Clinical research stage (multiple clinical studies, including RCTs), as a supplementary or alternative therapy for refractory patients.	Early clinical trial stage (Phase I/II), for relapsing/refractory NMOSD patients.

**Table 2:** Clinical trials using different cells for the treatment of NMOSD or MS.

Clinical trial number	Cell type	Autoimmune disease	Phase	Recruitment status	Estimated enrollment	Country	Start date
NCT02249676	AMSC	NMOSD	IIa	Completed	15	China	2013
NCT01364246	UC-MSC	MS, NMO	I/II	Unknown status	20	China	2010
NCT00787722	AHST	NMO	N/A	Completed	13	United States	2009
NCT01339455	AHST	NMOSD	N/A	Terminated	3	Canada	2011
NCT04561557	Anti-BCMA CAR T-cell	NMOSD	Early I	Recruiting	36	China	2020
ChiCTR-INR-16008037	hUC-MSC	NMOSD	I/II	–	–	China	2016

AMSC, autologous mesenchymal stem cell; UC-MSC, umbilical cord mesenchymal stem cell; AHSC, autologous hematopoietic stem cell; Anti-BCMA CAR T-cell, targeting mature B cell antigen chimeric antigen receptor T cell; hUC-MSC, human umbilical cord mesenchymal stem cell.

## 4 Other stem cell therapies

Neural stem cells (NSCs) demonstrate differentiation potential into oligodendrocytes, facilitating axonal myelination and neural regeneration. In murine models of neuromyelitis optica spectrum disorder (NMOSD), CFHR1-modified NSCs exhibit dual therapeutic effects: mitigating inflammatory infiltration and attenuating immune-mediated astrocytic damage. These modified NSCs achieve therapeutic efficacy through targeted inhibition of complement cascade activation, particularly by suppressing membrane attack complex (MAC) formation (70). While promising, comprehensive investigations regarding long-term therapeutic sustainability and biosafety profiles remain imperative. Oligodendrocyte progenitor cells (OPCs) possess inherent capacity for oligodendrocytic differentiation and myelin sheath reconstitution, positioning them as potential therapeutic agents for diverse central nervous system pathologies including Alzheimer’s disease (71, 72). Notably, clinical translation of OPC-based therapies in NMOSD remains unexplored, potentially limited by serum factor interference and age-dependent efficacy variations observed in patient populations (73). The current paucity of OPC-focused NMOSD research underscores a critical knowledge gap in this therapeutic domain.

## 5 Conclusion

In conclusion, cell-based therapies play a significant role in the treatment of NMOSD, particularly in patients with relapsing or refractory disease, and may even hold the potential to achieve a cure. Each cellular therapeutic approach possesses distinct advantages and limitations. Advancements in genetic engineering technologies, the prospects for cell therapy—especially CAR-T cell therapy—are considerable. To realize clinical potential, future investigations must prioritize: (1) multi-center randomized controlled trials with extended follow-up periods; (2) comprehensive safety assessments including off-target effects and cytokine release syndromes; (3) optimization of cell persistence and functional stability; and (4) mechanistic studies addressing therapeutic heterogeneity across patient subgroups.
